# Demonstration of anti-tumour bystander killing with prodrug-preloaded suicide gene-engineered tumour cells: a potential improvement for cancer therapeutics

**DOI:** 10.1186/s12935-020-1115-4

**Published:** 2020-01-28

**Authors:** Jehad Zweiri, Stephen E. Christmas

**Affiliations:** 1 Department of Molecular Medicine, King’s College London, The Rayne Institute, 123 Coldharbour Lane, London, SE5 9NU UK; 2 Department of Clinical Infection & Immunology, Institute of Infection & Global Health, University of Liverpool, Ronald Ross Building, 8, West Derby St, Liverpool, L69 7BE UK

**Keywords:** Tumour cell lines, Suicide gene therapy, Anti-tumour immune response, Cell death, Ganciclovir, Bystander killing effect, T cell immunosuppression and cancer clinical trials

## Abstract

**Background:**

Therapeutic approaches for cancer rely on careful consideration of finding the optimal way of delivering the pro-drug for cellular-based cancer treatment. Cell lines and cell cultures have been used in these studies to compare the in vitro and in vivo efficacy of autologous vs. allogeneic tumour cellular gene therapy. Here we have investigated and are reporting for the first time the effect of prodrug ganciclovir (GCV)-preloading (pre-treatment) in suicide gene therapy of cancer.

**Methods:**

This study examines the effect of GCV-preloading (pre-treatment) on a range of tumour cell lines in conjunction with suicide gene therapy of cancer. To determine the efficacy of this modality, a series of in vitro and in vivo experiments were conducted using genetically modified and unmodified tumour cell lines.

**Results:**

Following co-culture of herpes simplex virus thymidine kinase (HSV-TK) modified tumour cells and unmodified tumour cells both in vitro and in vivo, GCV-preloading (pre-treatment) of TK-modified human and mouse mesothelioma cells and ovarian tumour cells allowed them to mediate efficiently bystander killing of neighbouring unmodified tumour cells in vitro. In contrast, GCV-preloading of TK-modified human and mouse mesothelioma cells and ovarian tumour cells abolished their in vivo ability to induce bystander killing of unmodified tumour cells, although there was some tumour regression compared to control groups but this was not statistically significant. These results suggest that preloading TK modified tumour cells with GCV needs further study to define the most effective strategy for an in vivo application to retain their bystander killing potential after exposure to lethal doses of GCV in vitro.

**Conclusions:**

This study highlights the promising possibility of improving the efficacy of pro-drug system to prevent any damage to the immune system and enhancing this type of suicide gene therapy of cancer, as well as the need for further studies to explore the discrepancies between in vitro and in vivo results.

## Background

The prodrug-suicide gene therapy modality as applied for cancer therapeutics holds the potential to kill the tumour cells while triggering no collateral impairment to healthy cells [1, 2]. For example, the insertion of the herpes simplex virus thymidine kinase (HSV-TK) gene into tumour cells which are subsequently induced to “commit suicide” when in the presence of a non-toxic dosages of ganciclovir (GCV) [3, 4]. This careful selective toxic effect of the purine analogue ganciclovir is because HSV-TK phosphorylates ganciclovir, converting it eventually to ganciclovir-triphosphate, a very toxic compound when introduced into the DNA of these transfected tumour cells [5–8]. Moreover, it has also been established that two forms of “bystander tumour cell killing” mechanisms are mediated by this method: (a) a local “direct” bystander effect, as a result of the transfer of ganciclovir triphosphate from HSV-TK-positive tumour cells into untransfected neighbouring tumour cells [9–11], (b) a non-local systemic immunologically-mediated bystander effect due to the in vivo immune stimulation/presentation of tumour-specific or associated antigens following the killing of HSV-TK-expressing tumour cells [12, 13].

Moreover, it is well established that ganciclovir (GCV) causes bone-marrow toxicity in CMV-infected patients, particularly on the neutrophil lineage [14]. Therefore it may also induce T cell immunosuppression, although this does not appear to have been directly investigated. If GCV does have such a side-effect it may reduce the efficiency of the immunological component of the bystander effect induced by HSV-TK/GCV which have been reported by many groups [15, 16]. The rationale for the studies described here was to devise a strategy whereby TK+ve tumour cells would be exposed to GCV in vitro, in order to pre-load the tumour cells with GCV, wash the excess GCV away and then inject the cells for study of their in vivo bystander effect. It is also possible that the intravenous administration of GCV does not allow the achievement of a therapeutically high enough dose at the site of injection of TK+ve cells (e.g. in the peritoneum). By contrast, the pre-loading of TK+ve tumour cells with GCV may ensure that the cells have received the required dose of GCV. This may reduce the possible immunotoxic effects of GCV. This in turn may enhance the systemic immune mediated anti-tumour efficacy of treatment with HSV-TK expressing tumour cells. In this study we have shown for the first time to our knowledge the effect of GCV preloading (pre-treatment) on the fate of the bystander killing of TK-modified tumour cells, both in vitro and in vivo as well as possible ways to improve its action.

## Methods

### Cell lines

The human ovarian tumour (teratocarcinoma) cell lines PA-1 and PA-STK were obtained from Prof. S Freeman, Tulane University Medical School, New Orleans, USA [16]. Human mesothelioma cell lines CRL-5820, and 5830 were obtained from the American type culture collection (Rockville, MD, USA) with the permission of Prof. A Gazdar (MD Anderson Cancer Centre, Texas, USA) [17]. Mouse mesothelioma cell lines ABI (H-2d) from BALB/c mice, AE17 (H-2k) from CBA mice, and AC29 (H-2b) from C57BL/6 mice were obtained from Prof. B. Robinson, QEII Medical Centre, University of Western Australia, Nedlands, Australia. All human cell lines were maintained in DMEM, supplemented with 10% Fetal Calf Serum (FCS) and 1% sodium pyruvate. In the case of mouse mesothelioma cells, the RPMI-medium was supplemented with 5% FCS, 20 mM HEPES, 50 mM 2-mercaptoethanol (2ME) and 2 mM glutamine.

### Retroviral infection

Cells to be infected were seeded at a density of 5 × 10^5^ cells per 10 cm^2^ plate, 24 h before infection. Virus producing cells were also grown to about 90% confluence, and the medium changed 10 h prior to harvest of the culture medium to ensure that fresh virus containing supernatant was used [18].

The medium from virus producing cells was removed and filtered through a 0.45 μm pore-size filter to remove cell debris but allowing passage of the viral vector through the filter. To enhance the retroviral infection, 8 μg/ml of polybrene (Sigma Aldrich, Poole, UK) was added to the culture medium. Polybrene is required for coating of target cells in order to neutralise their negative surface charge, therefore increasing the efficiency of infection [19, 20]. The medium from the cells to be infected was removed prior to infection, and the vector-polybrene mixture added. After 10 h of infection the medium was changed. 48 h after infection, the medium was removed and replaced with fresh medium containing G418 at 1 mg/ml. G418 resistant clones were expanded.

### In vitro GCV-sensitivity studies on the tumour cell lines

The in vitro studies examining the effect of GCV on HSV-TK expressing mesothelioma and ovarian carcinoma cells, as well as the untransduced cells, were performed in 96-well plates. Transduced and untransduced cells were plated in triplicate at two densities, 10^4^ cells/well and 10^5^ cells/well for comparison. After 2 days, the medium was replaced by fresh DMEM containing the indicated concentrations of GCV in the range 10,000 μM to 0.01 μM. Cells were then incubated at 37 °C in a humidified 5% CO_2_ incubator for 5 days. Sensitivity to GCV treatment was measured by using a colorimetric cell proliferation assay that measures viable cell dehydrogenase activity, the microculture tetrazolium cell proliferation assay [21]. 20 μl of 5 mg/ml MTT (Sigma Aldrich, Poole, UK) was added to each well for 3 h and the cells in each well were solubilised in 150 μl of MTT solubilisation solution. After overnight incubation, the optical density of each well was measured on a 96-well plate reader (Dynatech, Reading, UK) set at 570 nm wavelength. Known concentrations of cells were also plated, cultured in the presence of MTT, and similarly solubilized. The absorbance reading of these control cells represents the metabolic activity of a known number of cells and was used to generate a standard curve in Microsoft Excel. The absorbance reading for each sample (well) was directly compared with the standard curve, and the numbers of viable cells were determined accordingly.

### Bystander killing effect studies

The bystander effect was determined by mixing HSV-TK expressing cells with untransfected cells at the indicated ratios. Cells were then plated in triplicate in 10 cm^2^ plates at two densities, 1 × 10^5^ and 5 × 10^5^ cell/plate to ensure cell–cell contact and to compare the in vitro effect of cell densities on the bystander effect. Two days later, the cells were treated with 50 μM GCV and incubated at 37 °C, 5% CO_2_ for 10–14 days. The plates were then stained with 2% methylene blue and stained cells were counted.

In order to calculate the effect of GCV on the mixed PA-STK and PA-1 populations, the number of colonies counted were expressed as a percentage of the total number of colonies in the co-cultured TK+ve and TK−ve tumour cells at the indicated ratios of the two cell populations in the presence of the indicated concentrations of GCV. A graph was obtained by plotting the percentage of surviving colonies.

### In vivo studies: inoculation, establishment and treatment of the tumours intraperitoneally (IP) in mice

AB1 mouse adherent mesothelioma cells were harvested by washing with versene only and re-suspended in 0.2 ml of PBS. Tumours were established IP in female BALB/c mice by injecting the mesothelioma cells (AB1 tumour cell line), at different doses (1 × 10^5^, 5 × 10^5^ and 1 × 10^6^/100 ml PBS) using a 26-gauge needle, to establish the TD_50_ for the tumour cell line, according to Home Office regulations. Mice (n = 25) were injected i.p. with AB1 tumour cells on day 0. Nine days later, animals were assigned to nine groups (n = 5 per group). GCV treatment was started at day 10. GCV (Cymevene® 500 mg; Roche, Switzerland) was diluted in sterile DMEM to a stock concentration of 50 mg/ml. The stock solution of GCV was diluted in DMEM to a concentration of 2 mg/ml, and 1 ml of the stock was injected IP once a day for five consecutive days. Mice were monitored every 2 days to palpate the tumour. At post-mortem, all tumour nodules were counted and measured using a calliper (for each nodule, 2 perpendicular diameters were recorded). Tumour volume was calculated for each nodule assuming spherical shape and the total tumour volume was calculated by adding all the calculated values for each mouse.

### Statistical analysis

Statistical analysis was performed using the Microsoft Excel program. Differences between groups were analysed using Student’s paired t-test. A p value of > 0.05 was considered as significant.

## Results

### GCV incubation time needed for the induction of cell death in PA-STK cells

**Fig. 1:**
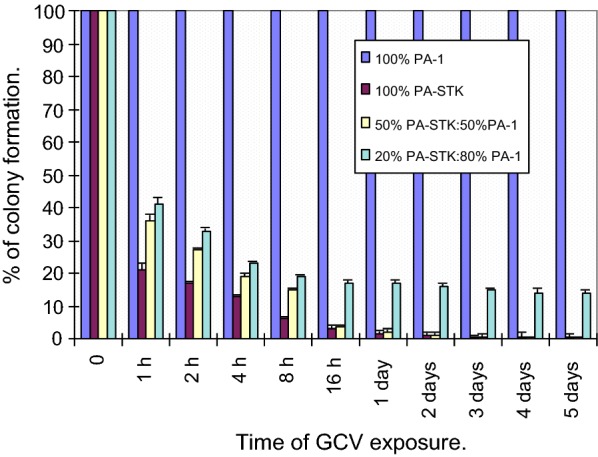
GCV incubation time needed for induction of cell death in PA-STK cells. Cells were treated with 50 μM GCV for the indicated time periods. The plates were then washed and fresh culture medium added without GCV for the rest of the experiment (total of 6 days). Survival was assessed by colony counting on day 6. Each point represents the mean of triplicate assays and error bars indicate standard error of the mean

This experiment was set up to determine the minimum time of GCV treatment required to induce cell death in the TK+ve cells. PA-STK cells were treated with 50 μM GCV for the indicated time periods. The plates were then washed and the medium was replaced with culture medium without GCV for the rest of the experiment (total of 6 days). Survival was assessed by colony counting as described before. It was concluded that 16 h of treatment was enough to reduce the number of colonies by about 95% (Fig. 1).

### GCV incubation time needed to induce the bystander effect

A similar experiment was conducted using mixed populations of PA-STK and PA-1 at 2 ratios 50:50 and 20:80, in order to assess the minimum time of GCV exposure to fully induce bystander killing of the TK−ve tumour cells. Figure 1 shows that the presence of GCV was required only in the first 16–24 h and that further prior exposure to GCV made little contribution to the subsequent bystander killing. These findings were reproduced in three independent experiments. Interestingly, even after 1 h GCV incubation, a significant bystander killing was detectable resulting in about 50% reduction in the number of colonies which were subsequently formed.

### GCV pre-treated PA-STK cells trypsinised and then co-cultured with PA-1 cells, do not mediate an in vitro bystander killing effect

**Fig. 2:**
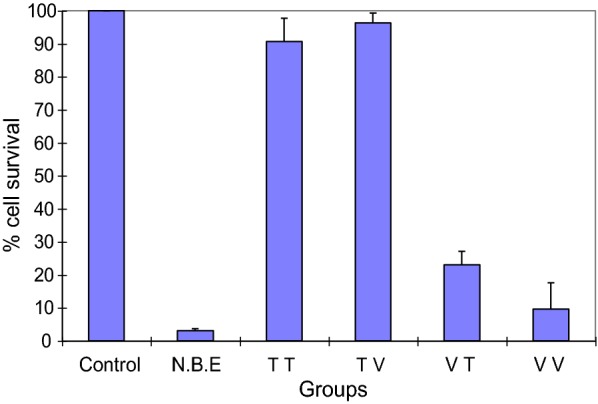
Versenised-GCV pre-treated PA-STK cells but not trypsinised cells, induce bystander killing. PA-STK cells were cultured with 50 μM GCV for 16 h and then either harvested by trypsin treatment or by versene treatment with no use of trypsin. They were then mixed with either trypsinised or versenised PA-1 cells in suspension and then plated for analysis of bystander killing by the MTT assay 5 days later. Control refers to the PA-STK and PA-1 cells mixed but not exposed to GCV. N.B.E, is the normal bystander effect resulting from co-culture of PA-STK and PA-1 cells in the continuos presence of GCV. TT refers to trypsinised PA-STK mixed with trypsinised PA-1 cells. T V refers to trypsinised PA-STK mixed with versenised PA-1. VT indicates versenised PA-STK mixed with trypsenised PA-1. VV indicates versenised PA-STK mixed with versenised PA-1. The total number of cells was 5 × 10^5^/well of 96-well tissue culture plate. In all mixing experiments the ratios of PA-STK to PA-1 cells was 50% and the GCV concentration was 50 μM. Error bars represent standard error of the mean. Representative of three similar experiments

Previous studies (above) had suggested that 16 h of GCV pre-treatment is sufficient for subsequent promotion of the bystander killing. PA-STK cells treated for 16 h with GCV, trypsinised, and then re-plated with PA-1 cells did not induce a bystander effect on the PA-1 cells (Fig. 2, TT or TV). Increasing the total number of cells in the plate (from 5 × 10^5^ to 1 × 10^6^) did not restore the bystander effect. Similarly reducing the time of GCV treatment to 8 h did not restore the bystander effect (data not shown).

In a parallel experiment PA-STK cells were treated with GCV for 16 h and then either trypsinised or versenised. PA-1 cells were also incubated with culture medium alone for 16 h and then either trypsinised or versenised. Mixing experiments were carried out with all 4 possible combinations (Fig. 2 and Table 1). The two different harvesting procedures were examined in order to find out whether trypsinisation inhibits the bystander killing by inhibiting the formation of gap junctions between PA-1 and PA-STK cells; this could be expected to affect the gap-junctional transfer of GCV-PPP. Touraine and his colleagues have shown that the magnitude of the bystander effect appears to correlate with the cell to cell transfer of phosphorylated GCV from the TK+ve cells to the adjacent wild-type cells [22].

**Table 1:** Effect of trypsinisation versus versenisation on bystander killing

PA-STK	PA-1	Bystander effect
Trypsin	Trypsin	−
Trypsin	Versene	−
Versene	Trypsin	+
Versene	Versene	+

Results in Fig. 2 are summarised in Table 1. This data shows that only the trypsinisation of the PA-STK cells (i.e. the cells which provide GCV-PPP) inhibited the bystander effect whereas trypsinisation of the target cells, PA-1, did not affect the bystander killing effect. This difference between the trypsinisation of the GCV treated cells rather than the “target” bystander cells may be due to the impaired metabolic activity of the GCV treated TK-positive cells. This would be compatible with the fact that these cells will be dying and therefore less able to restore their trypsin-damaged gap-junctions, whereas the TK−ve bystander cells would be able to re-synthesise their damaged gap-junctions. In vitro the TK−ve cells will be killed only if they had re-constituted their gap-junctions allowing the transfer of phosphorylated GCV into these cells.

PA-STK cells were treated with 50 μM GCV for 16 h and then either trypsinised or versenised, and mixed with either trypsinised or versenised PA-1 cells. The total number of cells was 5 × 10^4^/well of 96-well tissue culture plate. At the end of the treatment period, % survival was measured using MTT assay. The presence of bystander killing effect is shown in the table as (+), and the absence is shown as (−).

**Fig. 3:**
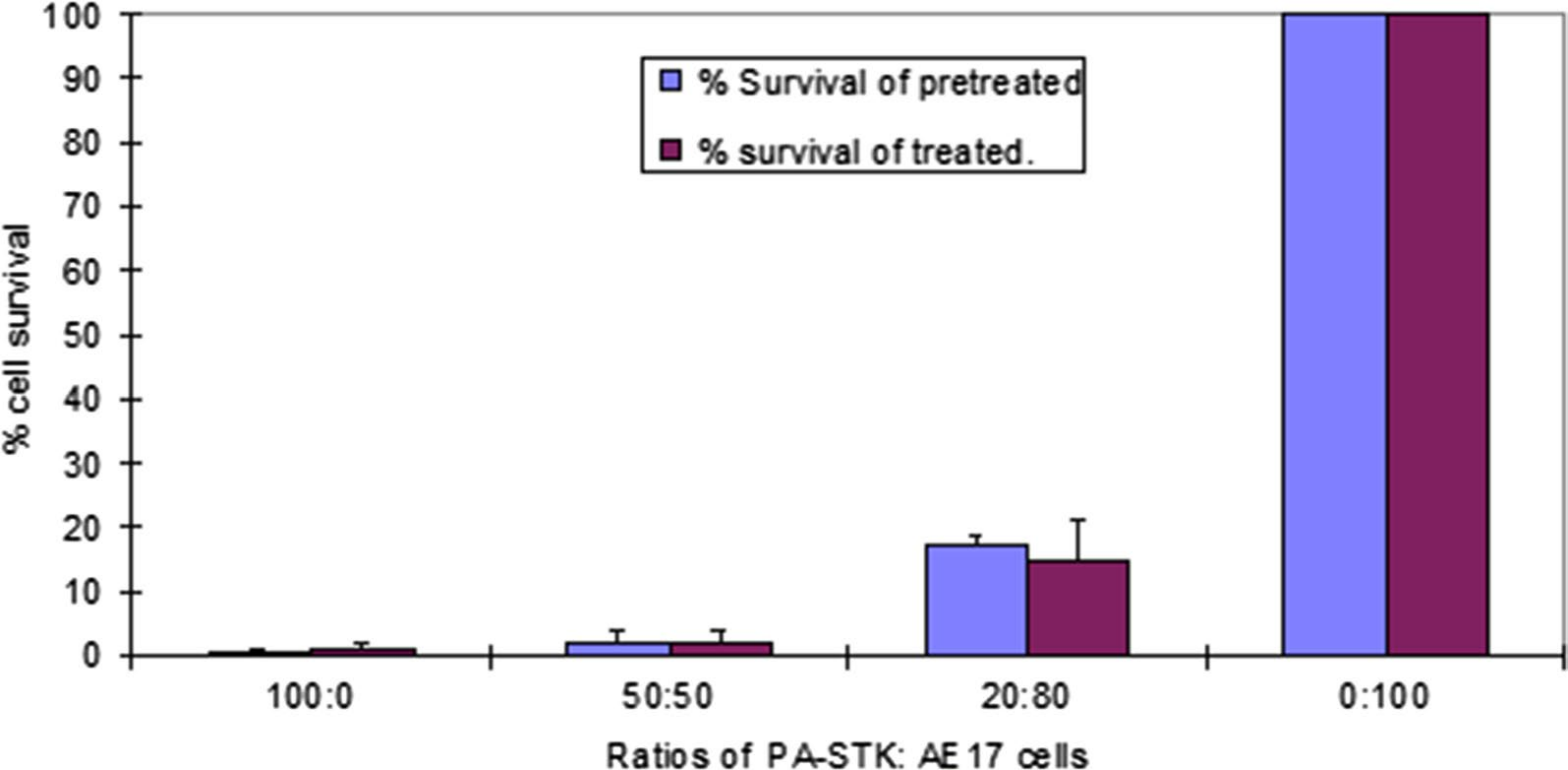
Versenised GCV pre-treated PA-STK cells induce bystander killing of the mouse mesothelioma cells AE17. PA-STK cells were pre-treated for 16 h with 50 μM GCV and then harvested by versene and cultured with AE-17 cells at the indicated ratios of PA-STK cells to AE17 cells, respectively. % cell survival was assessed by MTT assay in 96 well plates. Error bars represent standard error of the mean. Representative of three similar experiments

The next objective was to ascertain whether preloaded versenised PA-STK cells can induce bystander killing on cells other than PA-STK cells. Versenised GCV pre-treated PA-STK cells also induced bystander killing of mouse AE17 mesothelioma cells (Fig. 3).

**Fig. 4:**
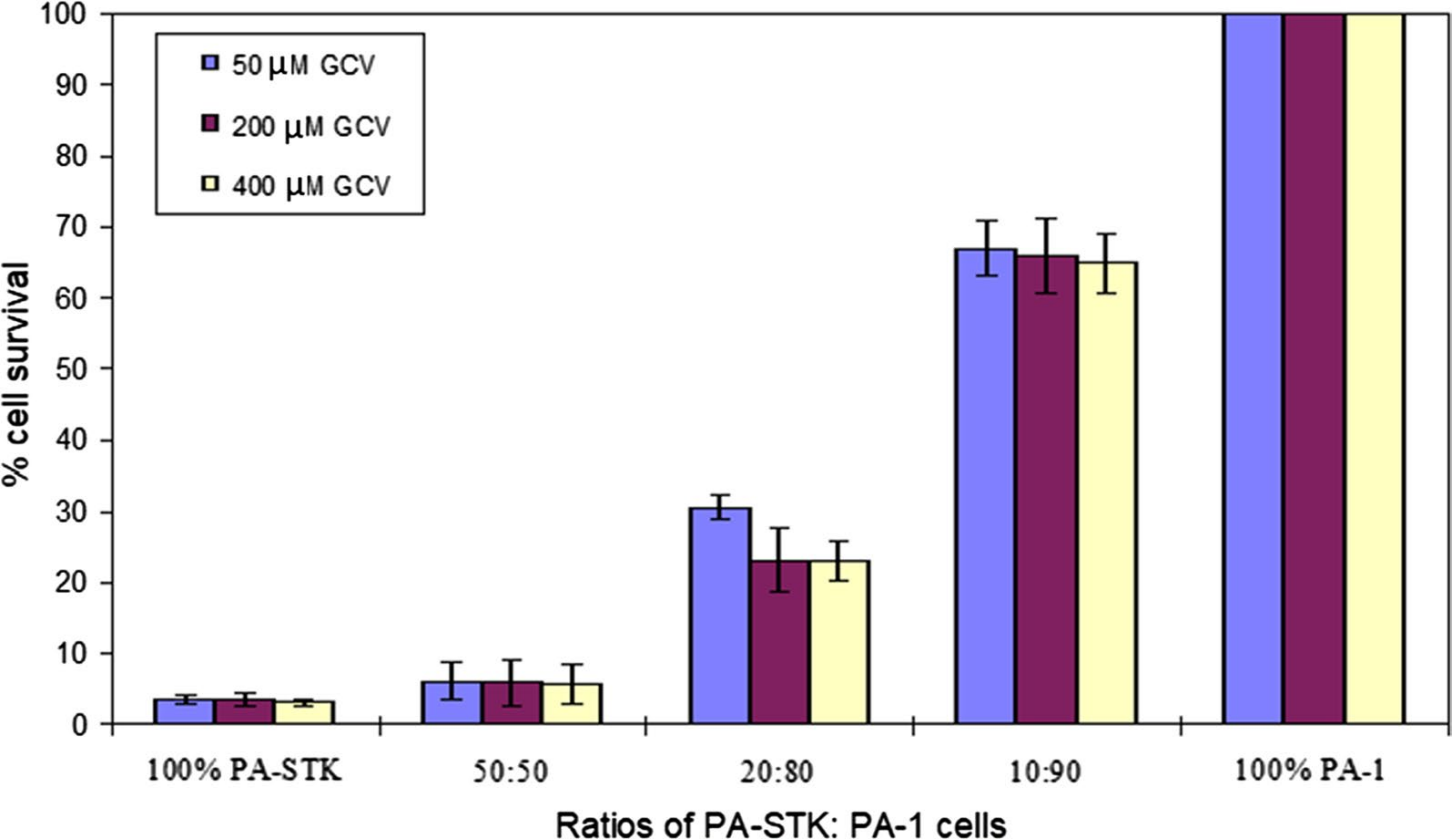
Effect of GCV concentration for the pre-loading of PA-STK cells on the bystander killing of PA-1 cells. PA-STK cells were pre-treated with different concentrations of GCV (μM) for 16 h. Cells were then harvested by versenisation, mixed and then incubated at the indicated ratios for 5 days.  % cell survival was assessed by the MTT assay in 96 well plates. The assays were performed in triplicate and error bars indicate the standard error of the mean. Error bars represent standard error of the mean. Representative of three similar experiments

The effect of GCV concentration during the pre-loading was next examined on the efficiency of the subsequent bystander killing by these preloaded cells. Increasing the GCV concentration up to 400 μM did not augment the bystander killing effect as shown in Fig. 4.

### Versenised pre-treated AE-STK cells can induce bystander killing of AE17 and AB1 cells

**Fig. 5:**
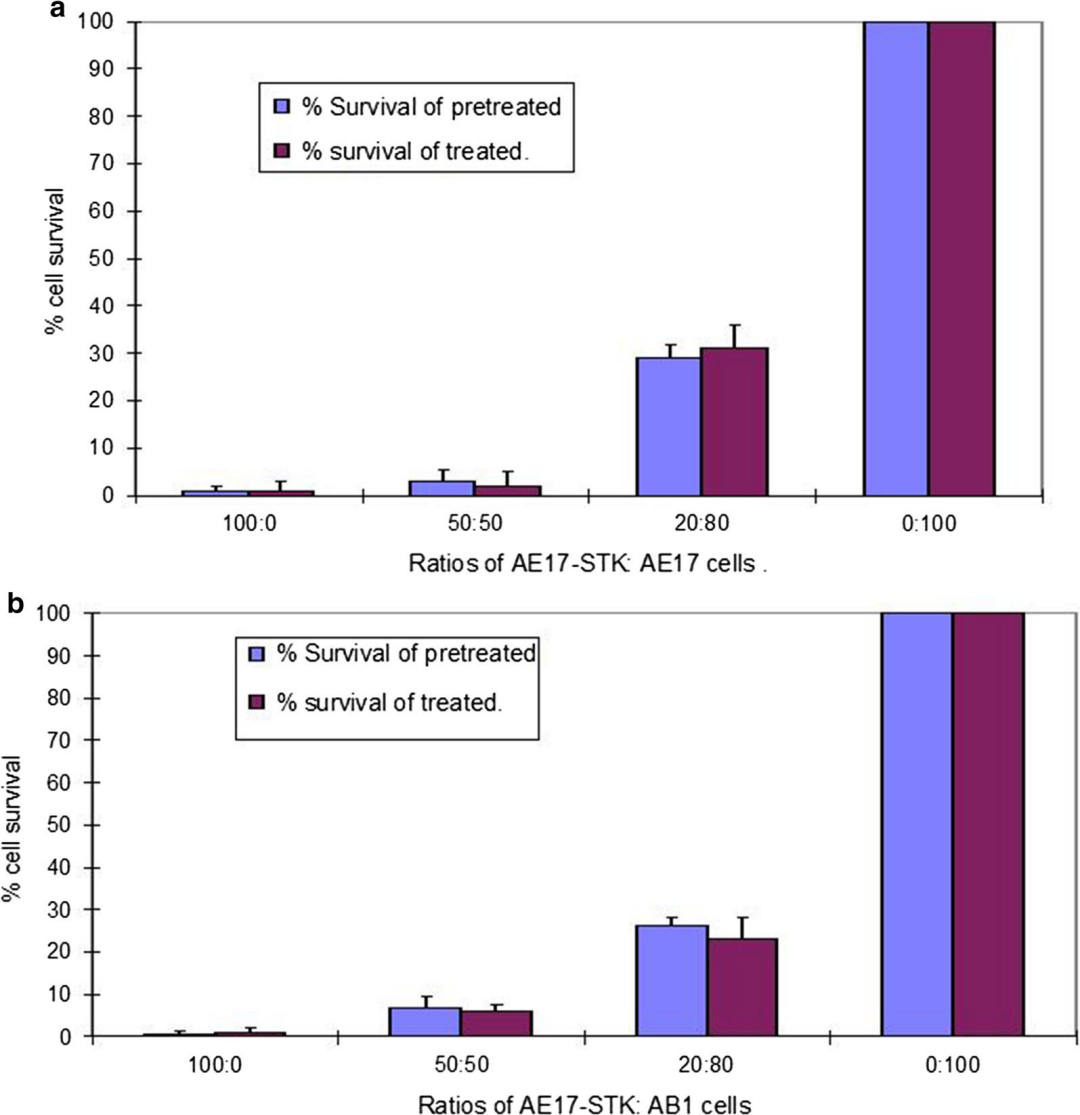
Pre-treated mouse mesothelioma AE17-STK induce bystander killing on: AE17 (**a**) and AB1 cells (**b**). The indicated ratios of AE-STK cells were mixed and plated at an initial cell density of 5 × 10^4^/well in 96-well plates. In one set the AE-STK cells were GCV pre-loaded (pre-treated) by incubation with 30 μM of GCV for 16 h. In the other set (treated) there was no GCV pre-loading, but 30 μM GCV was added to the culture for the duration of the experiment. These cultures were incubated for 5 days and % cell survival was assessed by the MTT assay. Error bars represent standard error of the mean. Representative of three similar experiments

In similar studies the ability of GCV pre-treated AE-STK cells to induce bystander killing of mouse mesothelioma cell lines AE-17 and AB1 were examined. The results show that GCV pre-loaded AE-STK cells can induce bystander killing of the autologous AE17 cells (Fig. 5a), as well as the allogeneic AB1 cells (Fig. 5b).

### Versenised pre-treated human CRL-5830-STK cells induce the bystander killing of wild type CRL-5830 and CRL-5820 cells

**Fig. 6:**
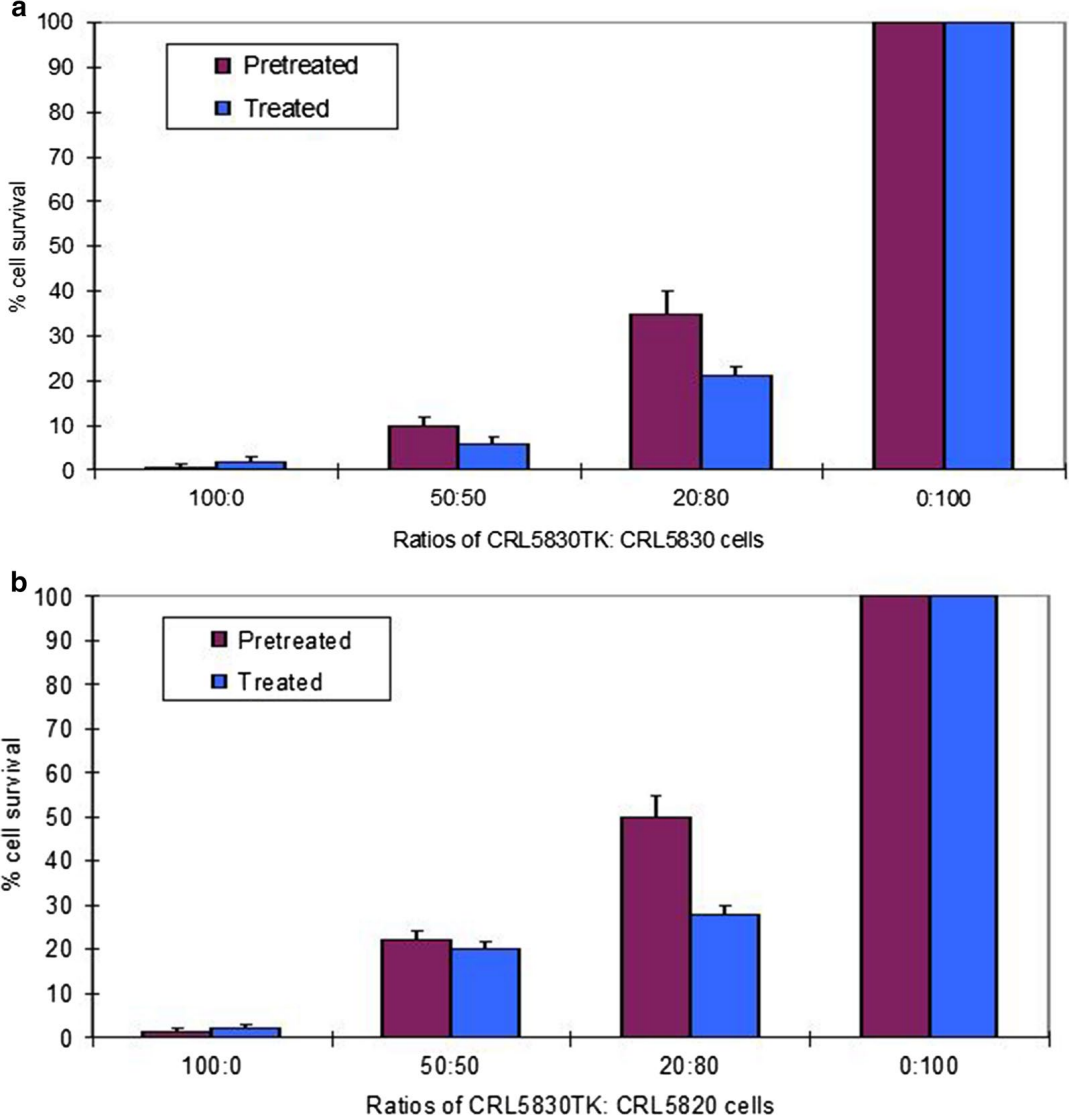
Pre-treated human mesothelioma CRL-5830-STK induce bystander killing of **a** CRL-5830 cells and **b** CRL-5820 cells. The indicated ratios of CRL-5830-STK cells were mixed and plated at an initial cell density of 5 × 10^4^/well in 96-well plates. In one set the CRL-5830-STK cells were GCV pre-loaded (pre-treated) by incubation in the presence of 30 μM of GCV for 16 h. In the other set (treated) there was no GCV pre-loading, but 30 μM GCV was added to the culture for the duration of the experiment. These cultures were incubated for 5 days and  % cell survival was assessed by MTT assay. Standard error of the mean is shown

Human CRL-5830-STK cells were pre-treated with 30 μM GCV for 16 h and were mixed with CRL-5830 or CRL-5820 cells in a 96-well tissue culture plate and incubated for 5 days before measuring cell survival by the MTT assay. The result of this experiment shows that GCV-pre-treated CRL-5830TK cells can induce bystander killing of both the autologous CRL-5830 cells (Fig. 6a), and the allogeneic CRL-5820 cells. However, at 20: 80 ratio, the bystander killing effect is not as efficient [p value = 0.012], when GCV is administered by pre-loading of the TK+ve cells (Fig. 6b).

The data presented have demonstrated that TK-modified tumour cells pre-treated (loaded) with GCV can induce the efficient bystander killing of unmodified tumour cells in vitro. The GCV incubation time needed to induce the bystander killing effect was also determined to be about 16 h, and this time was enough to result in subsequent death of over 90% of the pre-loaded TK+ve tumour cells. The data also show that versenised GCV pre-treated TK+ve tumour cells can induce the bystander killing of the TK−ve cells. However, trypsinisation of the GCV pre-treated TK+ve tumour cells inhibited the bystander killing of the TK−ve cells. Further increases in the GCV loading concentration of the TK+ve tumour cells did not augment their bystander killing of the TK−ve cells.

### Establishment of the allogeneic TK-tumour cells/GCV treatment in vivo model/preloading model

Mice (n = 25) were injected intraperitoneally with 1 × 10^6^ AB1 tumour cells in PBS on day 0. Nine days later (day 9), animals were assigned to five groups (n = 5 per group): group 1 received AE-STK cells + PBS only; group 2 received GCV only; group 3 received AE-STK + GCV (treatment group); group 4 PBS (control treatment group); group 5 received GCV-pre-loaded AE-STK cells as described below. On the same day (day 9), GCV treatment was started in group 2, 4 and 5 mice: 2 mg/ml GCV was injected intraperitoneally once a day for five consecutive days. Group 5, assigned as the GCV-preloaded group, were injected with the HSV-TK tumour cells (1 × 10^6^/mouse) previously incubated with 50 μM GCV for 16 h. These mice received no further GCV, but 2 mice in this group were given another intraperitoneal dose of GCV-preloaded TK-cells on day 12. About 6–7 days after the start of the treatment, mice in groups 2, 3 and 4 became unwell. They had greasy-looking coats and hunched backs.

**Fig. 7:**
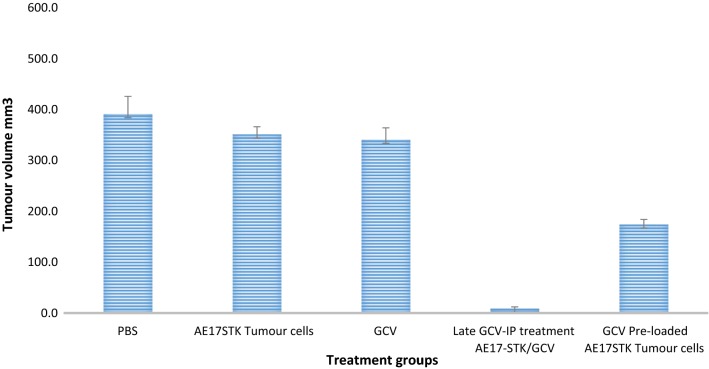
In vivo treatment using allogeneic AE17-STK mouse mesothelioma cells in BALB/c mice. BALB/c mice were injected IP with the syngeneic murine mesothelioma cells AB1 (1 × 10^6^ cells per mouse) on day 0. Nine days later the mice were separated into five treatment groups as indicated, and another healthy group acted as positive control. On day 18, mice were sacrificed and post-mortem was performed. All tumour nodules were counted and measured using a calliper. Tumour volume was calculated for each mouse and the mean tumour volume was calculated for each group. Error bars represent standard error of the mean. Representative of three similar experiments

On day 18, all the mice were sacrificed for post mortem examination. At post-mortem, all tumour nodules were counted and measured using a calliper (for each nodule, 2 perpendicular diameters were recorded). Tumour volume was calculated for each nodule assuming spherical shape and the total tumour volume was calculated by adding all the calculated values for each mouse. Those mice in the control group who received TK-modified tumour cells without GCV, all had significant tumours spread throughout the peritoneal cavity (100% tumour growth). The mice which received GCV alone for 5 doses intraperitoneally were found to have significant tumour growth in all mice out of five (100% tumour growth) (Fig. 7). The mice which were injected with the AE-STK cells and intraperitoneal GCV, were found to have no tumours (Table 2 and Fig. 7). This is a statistically significant reduction in tumour formation when compared to mice which received GCV alone or AE-STK cells, but no GCV [p value = 0.007]. However, mice which were injected with normal saline were found to be only 0% tumour free (i.e. 5 mice out of 5 were full of tumour). When compared to the group which received AE-STK + GCV, the difference was statistically significant [p value = 0.05]. However, in comparison to mice given either AE-STK or GCV alone there was a trend to significance [p value = 0.08]. All mice that were injected with the GCV-preloaded TK+ve tumour cells were found to have significant tumour growth except 2. Tumours were found throughout the peritoneal cavity and there was difference between this group and the control groups but was not significant (mice which received GCV alone or AE-STK alone) [p value = 0.65].

**Table 2:** In vivo treatment using allogeneic AE-STK cells against AB1 mesothelioma cells in syngeneic BALB/c mice

	Group 1	Group 2	Group 3	Group 4	Group 5
Treatment	AE-STK alone	GCV alone	AE-STK + GCV	PBS	GCV-preloaded
Number of lethargic/unwell mice	0/5	4/5	4/5	5/5	0/5

The apparent toxicity was evident only in the group of mice which received a regimen containing GCV (groups 2, 3, and 4). Interestingly, the mice in group 5, which received GCV pre-loaded AE-STK cells, did not show gross signs of toxicity as indicated by the absence of lethargy, greasy fur, or a hunched back posture.

Groups of mice (n = 5 per group) were challenged with 1 × 10^6^ AB1 mesothelioma cells on day 0. On day 9 they were given different treatments as specified on the top of the columns. On day 18, the mice were sacrificed and post-mortems were performed on all mice.

## Discussion

### TK-tumour cell GCV-preloading

It has been reported that the in vitro bystander killing is mediated over 3-days after GCV treatment [23, 24]. The data presented in this study has shown that the TK-expressing tumour cells need only a short incubation with GCV (16 h), to bring about almost 100% tumour cell death over the subsequent 3-days of culture without GCV. Based on this in vitro observation, we have postulated that the pre-treatment of TK-tumour cells with GCV may allow these cells to mediate bystander killing and tumour regression in vivo. Hamel and his colleagues have reported that 10 h incubation with GCV can be sufficient to induce cell death in most bystander cells co-cultured with HSV-TK expressing cells. However, in their study, time course of this effect was not formally examined [25]. Interestingly, their data suggest that most (if not all) of the bystander effect is mediated through gap junctions and not through phagocytosis of apoptotic vesicles. They argued that before 10 h no phagocytosis was visualised and therefore the apoptotic vesicles generated at a much later time points could not be the cause of cell death in the TK−ve bystander cells [8, 25].

The hypothesis tested was that this pre-loading protocol could allow the in vitro culture of the TK-modified tumour cells with high, clinically toxic dose of GCV in order to amplify the toxic bystander effect. Other protocols have also been developed by other groups in order to make sure that the right GCV concentration reaches the site of the tumour. In particular Engelmann and his colleagues have enhance the efficacy of HSV-TK bystander killing by using liposomal encapsulation of GCV, as a strategy to increase the GCV blood concentration at the tumour site [26].

In vitro experiments showed that GCV pre-loaded TK-tumour cells, trypsinised and then co-cultured with wild type tumour cells did not mediate bystander killing. However, when TK-tumour cells were versenised and then co-cultured with wild type tumour cells, they did mediate the bystander effect with efficiencies comparable to the usual continuous incubation in the presence of GCV.

It is most likely, but not proven, that the trypsinisation inhibits the bystander effect by degrading the gap junctions between the TK+ve and the bystander TK−ve cells. This would be compatible with the fact that these cells will be dying and therefore less able to restore their trypsin-damaged gap-junctions, whereas the TK−ve bystander cells would be able to re-synthesise their damaged gap-junctions. In vitro the TK−ve cells will be killed only if they had re-constituted their gap-junctions allowing the transfer of phosphorylated GCV into these cells. Blaese’s study showed that the bystander effect appears to correlate with the cell to cell transfer of phosphorylated GCV from the TK-expressing tumour cells to the adjacent wild type tumour cells [22]. They also showed that there were different phenotypes of cells in relation to sensitivity to the bystander effect: some cells were resistant whereas some cells showed a sensitive phenotype. The resistant phenotype appeared to be dominant since these “resistant” cells were not only resistant to bystander killing but were also inefficient in “donating” the bystander effect [27]. Although the degree of gap junctions in cells correlates with the bystander effect, some groups have reported that this was not so in specific systems e.g. with specific human colonic carcinoma cells [28].

The in vivo experiments using GCV pre-loaded TK-tumour cells did not show mediation of an anti-tumour effect as was shown in vitro. It is possible that the pre-loaded TK-tumour cells lose their ability to home on the tumour site. To date, there are no published reports of GCV-preloading experiments, in the context of HSV-TK/GCV suicide gene therapy. Preferential homing to the tumour site has been postulated by Freeman and his colleagues to explain anti-tumour activity seen after injection of TK+ve tumour cells in anatomical cavities which contain tumours [23]. Possible mechanism for the homing of the inoculated TK-gene modified tumour cells onto the tumour site include: (1) association of tumour cells more readily with tumour cells than with other non-malignant cells, (2) presence of tumour produced chemotactic factors [27–31] that attract other tumour cells and (3) in situ IP tumour masses may be devoid of a “repellent” mesothelial lining, thus allowing adherence of other cells [23].

## Conclusions

This study highlights the promising possibility of improving the efficacy of pro-drug system to prevent any damage to the immune system and enhancing this type of suicide gene therapy of cancer, as well as the need for further studies to explore the discrepancies between the in vitro and in vivo results. Also, a further future work may also address a repeat of those experiments, especially the in vivo ones, with another tumour model in order to see if that is a more general tumour-specific response or restricted to the unique model system described in this paper.

## Data Availability

The data and materials of this study are included in this published article.

## Abbreviations

GCV: ganciclovir
HSV-TK: herpes simplex virus thymidine kinase
MTT: micro-culture tetrazolium cell proliferation assay
